# Structural basis of human ABCC4 recognition of cAMP and ligand recognition flexibility

**DOI:** 10.1186/s13578-025-01377-y

**Published:** 2025-03-27

**Authors:** Xuepeng Wen, Kaixue Si, Dantong Zhu, Anqi Zhang, Changyou Guo, Minghui Li, Weiming Tian

**Affiliations:** School of Life Science and Technology, Harbin Institute of Technology, Harbin, China

**Keywords:** Structural biology, Cryo-EM, ABCC4, cAMP, 8-[Fluo]-cAMP, Transport

## Abstract

**Background:**

ABCC4 (ATP-binding cassette sub-family C member 4) is a transporter protein that is primarily localized to the plasma membrane, and its efflux activity is associated with the progression of various cancers and the development of drug resistance. Cyclic adenosine monophosphate (cAMP) is an important biomolecule that is considered a transport substrate of ABCC4. However, there is currently no direct structural understanding of how ABCC4 binds cAMP, and the mechanisms by which it recognizes a diverse range of substrate ligands remain poorly understood. Some studies have indicated that, under physiological conditions, cAMP does not significantly stimulate the ATPase activity of ABCC4, making the commonly used ATPase activity assays for ABC proteins unsuitable for studying cAMP.

**Results:**

Here, we successfully resolved the cryo-electron microscopy (cryo-EM) structure of the human ABCC4-cAMP (hABCC4-cAMP) complex, revealing how hABCC4 binds to cAMP and identifying the key residues involved. This structure was compared with two other hABCC4 complex structures we obtained (Methotrexate and Prostaglandin E_2_) and with previously published structures. We discovered some new structural insights into how hABCC4 binds ligands. On the basis of the structural information obtained, we confirmed the feasibility of using 8-[Fluo]-cAMP in a transport assay to detect cAMP translocation and found that some challenges remain to be addressed.

**Conclusions:**

These results suggest that hABCC4 can bind cAMP and exhibits varying degrees of flexibility when binding with different substrates, including cAMP. These findings expand our understanding of the structural biology of ABCC4.

**Supplementary Information:**

The online version contains supplementary material available at 10.1186/s13578-025-01377-y.

## Introduction

ATP-binding cassette (ABC) transporters are among the largest and oldest families of transporters discovered to date [1]. Human ABC transporters encompass seven subgroups, ranging from ABCA transporters to ABCG transporters [2], and these transporters are expressed in nearly all cells and tissues with varying degrees of abundance [3]. ABCC4 is a member of the ABCC family and is ubiquitously distributed across various tissues and cell types [4]. The ABCC4 protein primarily consists of four domains, namely, two transmembrane domains (TMDs) and two nucleotide-binding domains (NBDs) [5]. In addition, there is a shorter lasso motif at the N-terminus and a PDZ (PSD-95/Dlh/ZO1, a protein motif capable of recognizing and binding to specific types of protein sequences.) motif at the C-terminus [6]. Structurally distinct from other C-family proteins, ABCC4 lacks a TMD0 domain [7–11]. Furthermore, it does not possess an R-domain, similar to that found in ABCC7 (CFTR), which regulates its transport function through phosphorylation [12]. Within the domain structure of ABCC4, both TMDs play crucial roles in substrate recognition and binding processes [13]. ABCC4 can transport a diverse range of substrates [14, 15], and its involvement in numerous vital cellular physiological processes has been well documented [16–20]. Cyclic adenosine monophosphate (cAMP) is considered one of the substrates transported by ABCC4 [21] and plays a significant role in the development and progression of various cancers [22–24]. For example, when AML (Acute Myeloid Leukemia, a malignant hematologic tumor derived from myeloid progenitor cells) [25] cells are stimulated to produce intracellular cAMP through amthamine, inhibiting the ABCC4-mediated efflux of cAMP via specific inhibitors significantly reduces the efficiency of AML cell proliferation [26, 27]. However, a direct structural understanding of how hABCC4 recognizes and binds to cAMP is lacking.

Although numerous studies have demonstrated an association between ABCC4 and cAMP transport [28–32], limited research has been conducted on the direct binding and transport of cAMP through ABCC4. A study published in 2024 suggested that the effect of ABCC4 on cAMP may be attributed to its role as a transporter of various prostaglandins, thereby activating associated receptors and initiating a cascade of subsequent reactions, leading to alterations in intracellular cAMP levels rather than direct transport. In this study, cAMP failed to stimulate an increase in the ATPase activity of ABCC4 (*Bos taurus*) in vitro and remained at basal hydrolysis levels. The inability of cAMP to stimulate the ATPase activity of ABCC4 presents an additional challenge in studying the transport of cAMP by ABCC4 [33]. Notably, this phenomenon extends beyond cAMP, as other substrates of ABCC4, such as cyclic guanosine monophosphate (cGMP), exhibit similar behavior [33]. Although alternative methods, such as the reconstituted vesicle transport assay and radioactive tracer assay [34–37], are available, these approaches are highly complex, costly, and require significant technical expertise, making them inaccessible to most researchers. This greatly limits the study of the interaction between ABCC4 and cAMP. Therefore, developing a low-cost and accessible method for detecting cAMP would be of significant practical value and provide a valuable reference for studying cAMP and ABC family proteins, including ABCC4.

In the study of certain ABC proteins, researchers have attempted to use fluorescently labelled substrates for transport studies and have made some progress [38]. 8-[Fluo]-cAMP, a fluorescently labelled cAMP, has been suggested to be transported by hABCC4 in cellular transport experiments [39]. However, several issues remain to be addressed regarding the use of 8-[Fluo]-cAMP in the study of cAMP and ABCC4. First, while the efflux transport of 8-[Fluo]-cAMP in cellular experiments has been shown to be associated with hABCC4, this alone is insufficient to confirm that it is directly transported by hABCC4 or that it forms a complex with hABCC4. Second, 8-[Fluo]-cAMP and cAMP are not identical molecules. 8-[Fluo]-cAMP is a modified version of cAMP with a fluorescent group attached, which is relatively large compared with cAMP. This modification may have a significant effect on the molecular binding process with ABCC4, which cannot be ignored. Even if 8-[Fluo]-cAMP is assumed to be directly transported by hABCC4, it remains uncertain whether it binds to hABCC4 in the same manner as cAMP does. Furthermore, even if 8-[Fluo]-cAMP is directly transported by ABCC4, it is possible that hABCC4 recognizes and binds primarily to the fluorescent group rather than the cAMP portion of the molecule. Most importantly, owing to the lack of structural studies on the cAMP-hABCC4 protein complex, we still do not fully understand how hABCC4 binds to cAMP or the key amino acid residues involved. This makes designing targeted experiments to test whether 8-[Fluo]-cAMP is transported by hABCC4 on the basis of the structural information of cAMP challenging.

To address the issues caused by the lack of structural data, we obtained a high-resolution cryo-electron microscopy (cryo-EM) structure of hABCC4 in complex with cAMP. This structure revealed the key residues involved in cAMP binding as well as the specific characteristics of the binding interaction. To validate the accuracy of the cryo-EM sample preparation and structural data, we also resolved the complex structures of hABCC4 with two previously reported molecules, Methotrexate (MTX) and Prostaglandin E_2_ (PGE_2_), which are critical transport substrates of hABCC4 [40, 46]. Existing structural information (PDB IDs: 8BWP and 8BWR) [45] suggests that these molecules may illustrate the mechanism of ligand flexible recognition by hABCC4. These structures served as reference controls to validate the binding characteristics and structural integrity of the hABCC4-cAMP complex. Additionally, using structural information from the hABCC4-cAMP complex, we designed experiments to explore the potential feasibility of using 8-[Fluo]-cAMP as an experimental tool for detecting cAMP transport by hABCC4. Our study provides structural insights into how hABCC4 binds substrate molecules, including cAMP, and functional experiments on 8-[Fluo]-cAMP offer valuable references for the development of new methods to detect cAMP and ABC proteins.

## Results

### Biochemical characterization of hABCC4

The purity and physiological activity of hABCC4 protein are essential for acquiring high-quality cryo-electron microscopy (cryo-EM) data. Active ABC proteins exhibit low ATPase activity in the absence of substrates, which is known as basal ATPase activity. In the presence of specific substrates, the hydrolytic activity of active ABC proteins increases significantly beyond their basal ATPase activity, a phenomenon referred to as substrate-stimulated ATPase activity. PGE_2_, a key transport substrate of hABCC4, stimulates hABCC4 to achieve substrate-stimulated ATPase activity that significantly exceeds its basal ATPase activity during transport [41]. Although MTX also stimulates hABCC4, its effect is relatively weaker compared to that of PGE_2_ [41, 45]. Therefore, after purifying the hABCC4 protein, we measured its basal ATPase activity and substrate-stimulated activity in the presence of PGE_2_ to confirm its physiological activity and ensure the reliability of the complex structure determination process.

**Fig. 1:**
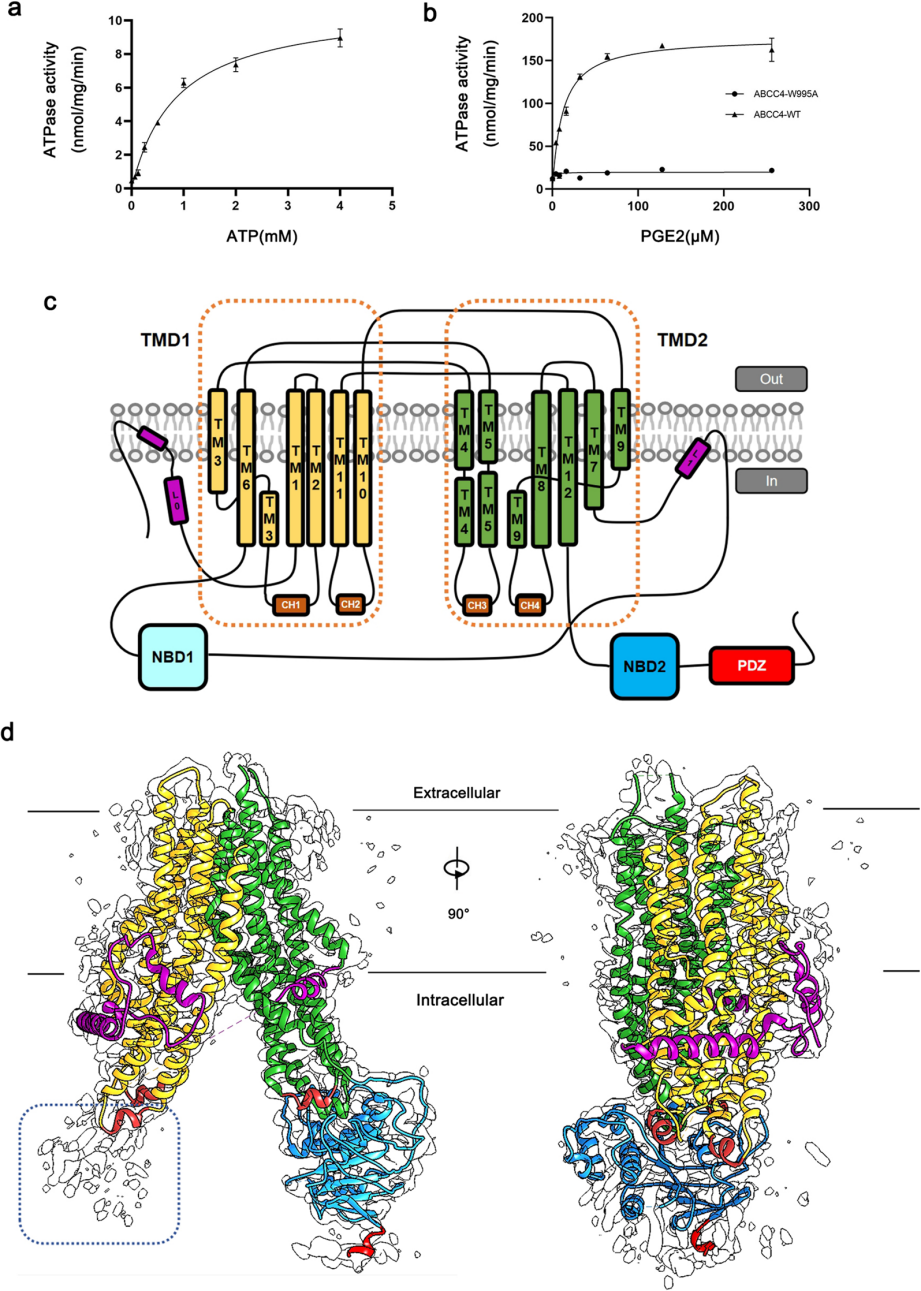
Overall schematic diagram of hABCC4 (Apo). **a** Basic ATPase activity of hABCC4-WT. The hABCC4 protein we obtained for cryo-EM sample preparation exhibited changes in hydrolytic activity with increasing ATP concentrations in the absence of substrates, which is consistent with the characteristics of physiologically active proteins (n = 5; data are presented as the means ± SEMs). **b** ATPase activity of hABCC4-WT and hABCC4-W995A in the presence of PGE_2_. In the presence of the substrate PGE_2_, the ATPase activity of the hABCC4-WT protein was significantly greater than its activity in the absence of substrate. However, this increase in ATPase activity was markedly attenuated when the key residue W995, which is responsible for PGE_2_ recognition, was mutated. This observation is consistent with the physiological characteristics of hABCC4 (n = 5; data are presented as the means ± SEMs). **c** Schematic representation of hABCC4. Twelve transmembrane helices are sequentially numbered from the N- to the C-terminus, resulting in TMD1 and TMD2. The NBD1 and NBD2 domains are responsible for ATP hydrolysis. L_0_ represents the lasso motif. The PDZ domain is located at the C-terminus. **d** hABCC4 in the apo state density map with refined models. The map reveals the structural integrity of the hABCC4 protein. The NBD1 domain has limited structural resolution, and considering its lack of relevance to the substrate-binding pocket, no structural model has been built for this domain (blue dashed line location)

The basal ATPase activity assay revealed that the hABCC4-WT protein exhibited ATP hydrolysis activity in the presence of ATP and Mg^2+^ within the LMNG detergent environment, with activity values consistent with the Michaelis‒Menten equation (K_m_ 0.087 mM, V_max_ 10.91 nmol/mg/min, Fig. 1a).

The stimulated hydrolytic activity of hABCC4 was observed in the presence of PGE_2_, ATP, and Mg^2+^. The experimental findings (Fig. 1b) revealed a significant increase in the hydrolytic activity levels (K_m_ 11.81 mM, V_max_ 177 nmol/mg/min) of hABCC4-WT when PGE_2_ was present compared with ATP alone. In contrast, the hydrolytic activity of hABCC4-W995A (one of the key residues involved in binding to PGE_2_) was only slightly greater (K_m_ 0.88 mM, V_max_ 19.75 nmol/mg/min) than that of hABCC4-WT basal ATPase activity. These findings suggest that PGE_2_ significantly enhances the hydrolytic activity of hABCC4. However, mutation at residue W995 substantially reduced this increase.

The results of the ATP hydrolysis assay demonstrated that the physiological activity of the purified hABCC4 protein was maintained in an in vitro detergent environment, confirming the reliability of the structural data obtained for hABCC4.

### Cryo-EM structure of the hABCC4-cAMP complex

#### Comparison of the substrate-binding pocket region between hABCC4-Apo and the three complex structures

To obtain a reliable structure of the hABCC4-cAMP complex, we also determined three additional protein structures in this study via the same sample preparation method used for hABCC4-cAMP: hABCC4 (Apo), hABCC4 bound to methotrexate (hABCC4-MTX), and hABCC4 bound to prostaglandin E_2_ (hABCC4-PGE_2_). The hABCC4-Apo (Fig. 1c, d) structure was used to illustrate the substrate-binding pocket of hABCC4 and the state of the pocket in the absence of substrate binding. The hABCC4-MTX and hABCC4-PGE_2_ complex models were compared with previously published structural models to ensure the reliability of the observed density of the cAMP ligand within the substrate-binding pocket in the hABCC4-cAMP structure. These measures were taken to ensure the reliability of the cryo-EM sample preparation method and the resulting structural data.

**Fig. 2:**
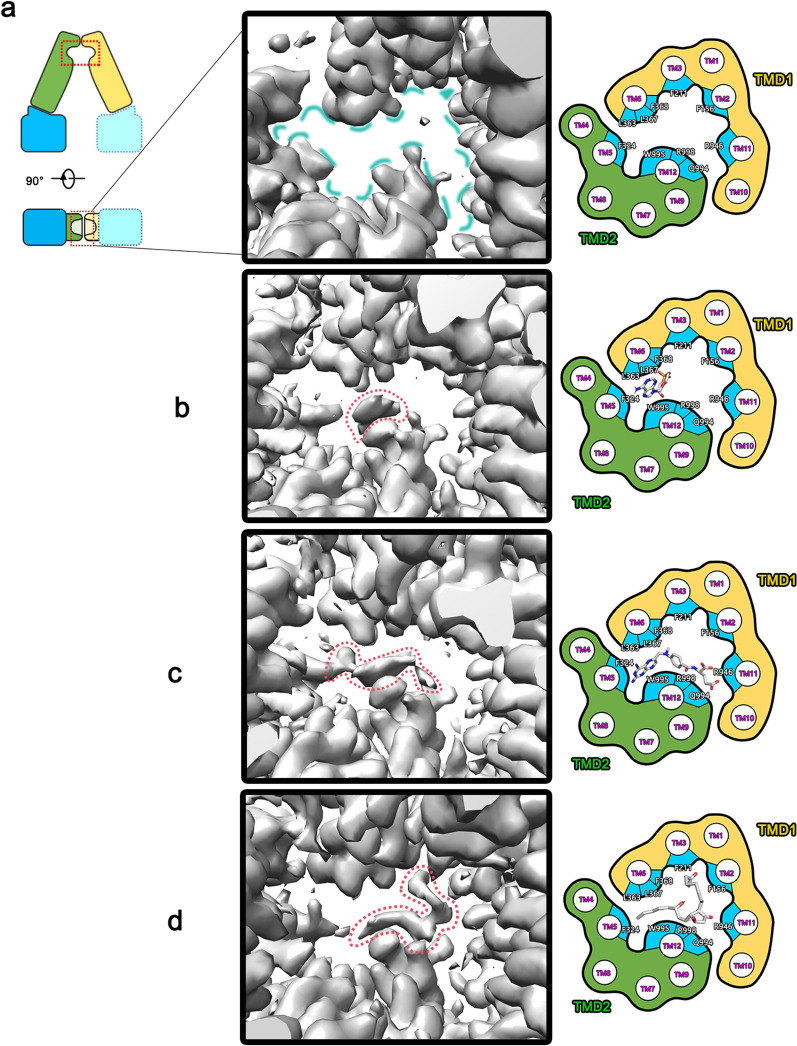
Structural diagram of the substrate binding region of hABCC4. Structural model illustrating the hABCC4 substrate pocket region. Schematic diagram (**a**) visually illustrates the Apo state pocket, which highlights the cavity in this region (blue dashed circle). **b**–**d** represent the pockets of the cAMP, MTX, and PGE_2_ complexes, respectively. The density of the ligands within the pockets is clearly visible, as indicated by the red dashed lines. The cartoon representations on the right provide a more intuitive visualization of the regions occupied by the ligands within the pockets

On the basis of the analytical structural data, the resolved hABCC4 protein exhibited an average resolution of 3.29 Å. Comparison of the density maps of the substrate-binding pocket between hABCC4 (Apo) (Fig. 2a) and the other three complex structures clearly reveals the density of the substrate molecules within the binding pocket in the three complex structures (Fig. 2b–d). These structures (Fig. 2 right side cartoon) revealed that the substrate-binding region of hABCC4 is a cavity formed by helices TM2, TM3, TM5, and TM6 in the TMD1 domain and helices TM9, TM11, and TM12 in the TMD2 domain. Helices TM2, TM3, TM5, TM6, TM9, and TM11 collectively form an approximately elliptical cavity surrounding the periphery, whereas helix TM12 traverses the central axis of the cavity. In the top-down view, the binding region appears as a fan-shaped pocket. The outer ring of the pocket is surrounded by residues F156, F211, F324, L363, L367, F368, and R946, whereas the center of the pocket contains residues Q994, W995, and R998 located in helix TM12. This finding is consistent with other reported hABCC4 [33, 43–45].

A comparison of the maps of hABCC4 (Apo) with the three complex structures clearly revealed the density maps of the substrate ligands within the binding pocket of hABCC4 in all three complexes. The obtained complex data can be used for further ligand-binding and molecular fitting analyses.

#### hABCC4-cAMP complex structure

An analysis of the density map revealed that although the overall resolution of the hABCC4-cAMP complex structure reached 2.99 Å, the density of the cAMP molecule within the substrate-binding pocket was not ideal. At a credible level (Chimera set level 0.480), the density of the cAMP ligand was clearly defined but was primarily concentrated around near the W995 residue and its adjacent regions (Fig. 2b). The electron density did not fully cover the entire molecule, resulting in suboptimal molecular fitting (Fig. 4a). We hypothesize that this unusual phenomenon may be due to the flexible recognition and binding of cAMP by hABCC4: part of the cAMP molecule is tightly bound, whereas other regions are loosely bound and exhibit a degree of mobility. The density of the loosely bound regions cannot be resolved, leading to the observed issue where the density does not fully accommodate the molecular model. This hypothesis will be further explored in the discussion section, specifically in the context of the flexibility of ligand binding by hABCC4.

**Fig. 3:**
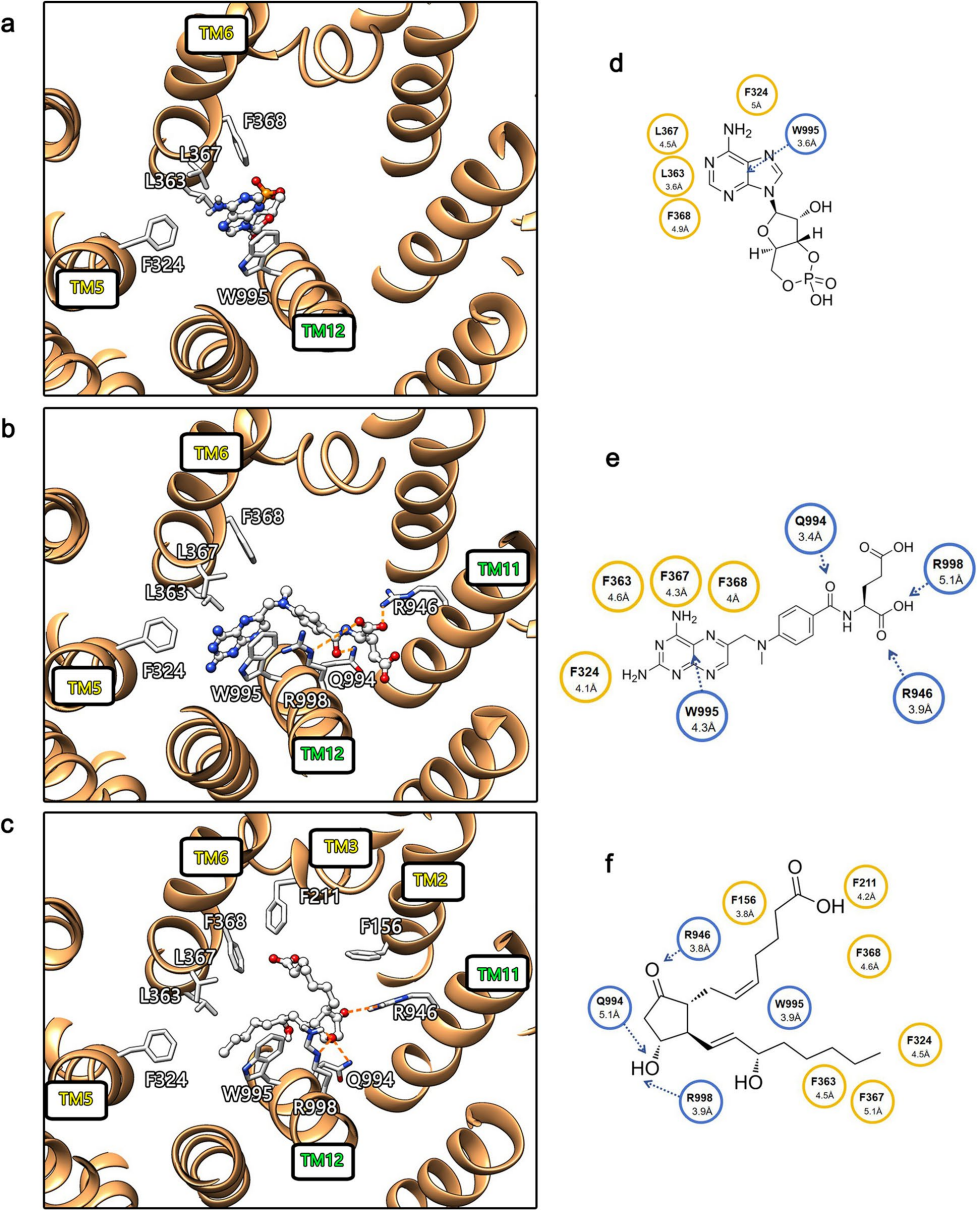
Schematic representation of the substrate‒ligand binding structure of hABCC4. The binding interactions between hABCC4 and ligands in the three complex models of cAMP, MTX, and PGE_2_ are shown. **a**–**c** depict the structural models of the binding pocket regions for the three complexes, where key residues are represented as sticks, ligands are shown as ball‒stick models, and directed interactions are indicated by orange dashed lines. **d**–**f** provide 2D representations of the ligands, with interaction distances labelled on potential interacting residues. Residues located in TMD1 are highlighted in yellow, those in TMD2 are highlighted in blue, and directed interactions are represented by dashed lines

Owing to the uncertainty in the density, our cAMP structural model was built on the basis of the maximum credible interpretation (Fig. 3a). The electron density of cAMP was predominantly localized around W995. Within this concave region, cAMP interacts with F324 from helix TM5, as well as with L363, L367, and F368 from helix TM6, along with W995 from helix TM12. Notably, hydrophobic contacts are formed between F324, L363, L367, and F368; the indole group of W995 interacts with cAMP through π‒π stacking interactions (Fig. 3d).

Although there may be some uncertainty in the construction of the structural model, the credible density of the cAMP ligand and the interaction types with relevant residues suggest that the hydrophobic interactions involving residues F324, L363, L367, and F368 may contribute to the binding of the hydrophilic cAMP molecule, albeit to a limited extent. However, the role of the W995 residue is uniquely distinct and critical.

#### Comparison of hABCC4-MTX and hABCC4-PGE_2_ with previously reported structures

We obtained the structures of hABCC4 in complex with MTX and PGE_2_. In addition to the reasons mentioned earlier, which ensured the credibility of the substrate ligand density in the cAMP complex and the cryo-EM sample preparation protocol, another purpose of obtaining these two independent complex models was to compare them with previously reported structural models (PDB IDs: 8BWP, 8BWR). This comparison aims to explore the potential flexibility in substrate binding by hABCC4. This work became particularly necessary after analysis of the hABCC4-cAMP structure revealed the possibility of flexible ligand binding for the cAMP molecule.

On the basis of the map data, we constructed two models, 9KRM and 9KRN (Fig. 3b, c). In the MTX complex 9KRM, MTX interacts with F324, L363, L367, F368, R946, Q994, W995, and R998. Among these interactions, F324, L363, L367, and F368 are hydrophobic, whereas R946 forms electrostatic interactions with MTX. Additionally, Q994 forms polar interactions with MTX, W995 interacts with MTX through π‒π stacking interactions, and R998 forms hydrogen bonds with MTX (Fig. 3e). In the PGE_2_ complex 9KRN, PGE_2_ interacts with F324, L363, L367, F368, F211, F156, R946, Q994, W995, and R998. PGE_2_ exhibits hydrophobic interactions with F156, F211, F324, L363, L367, and F368; forms polar interactions with Q994; forms electrostatic interactions with R946; and forms hydrogen bonds with R998 (Fig. 3f).

**Fig. 4:**
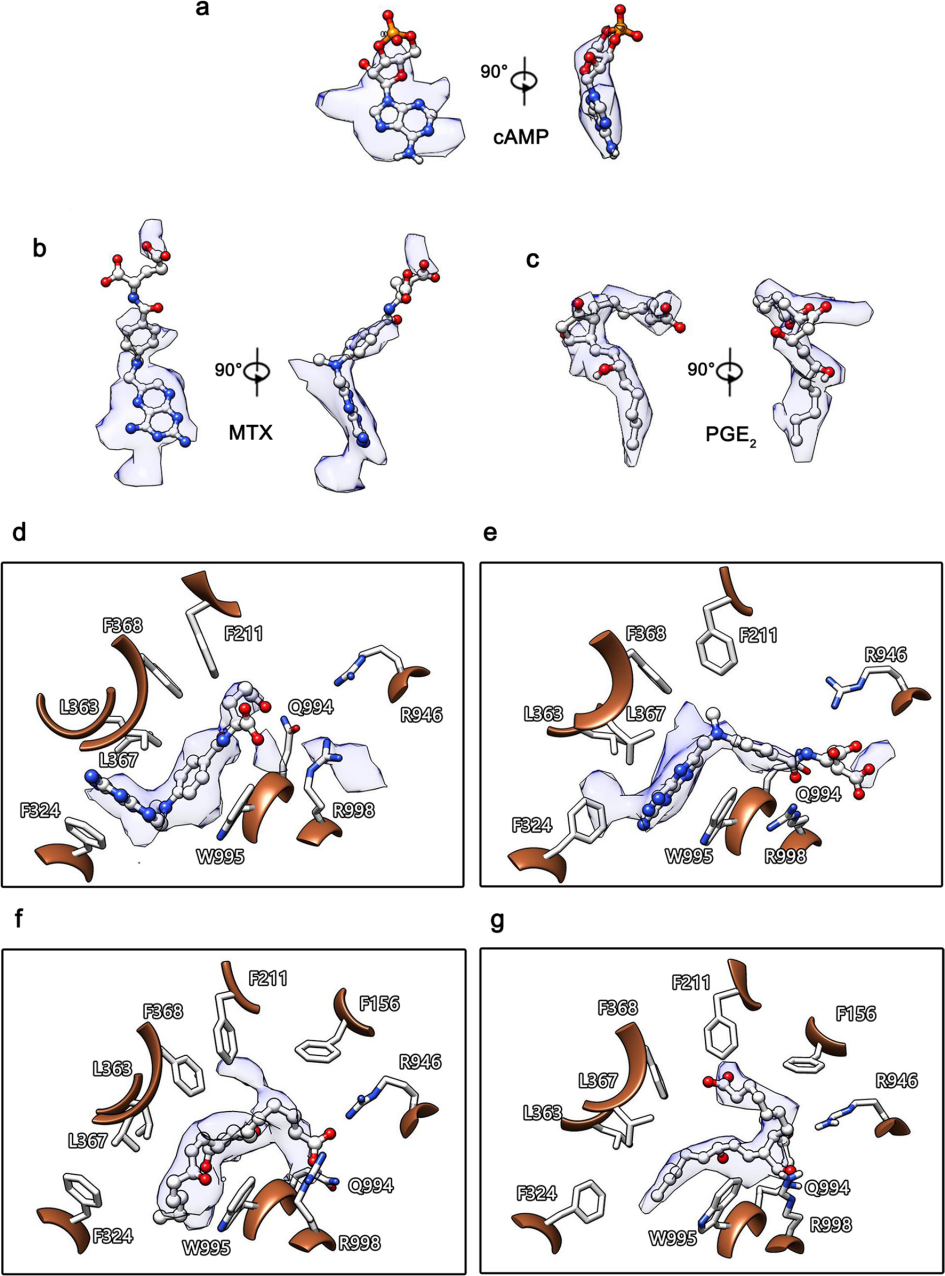
Comparison of the structures obtained in this study with those published from other sources. **a**–**c** illustrate the fitting of ligands into the density maps of the three hABCC4 complexes we obtained. The density coverage of PGE_2_ is relatively complete. In contrast, certain regions of MTX and cAMP exhibit poorer density coverage, indicating that the binding flexibility of these two ligands is greater than that of PGE_2_. **d**, **e** compare the MTX-bound structure we obtained (9KRM) with the previously published structure (8BWP). The density distributions of the MTX ligands in both structures are similar. However, in 9KRM, the ligand density in the W995-R998 region is continuous, suggesting that the sparse and dispersed density in this region may belong to MTX and that MTX exhibits high binding flexibility in this area. **f**, **g** compare the PGE_2_-bound structure we obtained (9KRN) with the previously published structure (8BWR). The density distributions of PGE_2_ in 9KRN and 8BWR are consistent and continuous, indicating low binding flexibility of the ligand

We compared the obtained maps with previously reported structural models. Compared with the reported maps of the hABCC4-MTX and hABCC4-PGE_2_ complexes (IDs: EMD-16293, EMD-16295), although different approaches were used for ligand modelling in our structures, the density distributions of the MTX and PGE_2_ ligands in our models exhibited fundamentally consistent patterns with the previously reported structures (Fig. 4d–g). The electron density of the ligand in the PGE_2_ complex map we obtained exhibited a clear overall contour and more complete coverage of the ligand molecule, resulting in an ideal molecular fit (Fig. 4c). These findings indicate that the hABCC4-PGE_2_ complex structure we obtained is reliable and that the cryo-EM sample preparation method used to derive the structural model is also robust. More importantly, the ligand density fitting results of PGE_2_ indicate that the binding of PGE_2_ by hABCC4 is highly specific and exhibits very low flexibility.

In both the map we obtained (EMD-62533) and the previously reported map (EMD-16293), the MTX ligand exhibited an uneven density distribution, characterized by clear and concentrated density in most regions but sparse and discontinuous density in certain areas. The map of EMD-16293 (Fig. 4d) shows relatively clear density for the MTX ligand near residues F324, L367, F368, and W995 within the substrate-binding pocket. Additionally, there is suspected MTX density near R998 and W995; however, this density is discontinuous, with density maps near F324, L367, F368, and W995. In contrast, the map we obtained (Fig. 4e) shows a similar electron density pattern for MTX near F324, L367, F368, and W995, but the density for MTX near W995 and R998 in our map is continuous (Fig. 4b). This suggests that the discontinuous density near R998 in the EMD-16293 structure may indeed belong to MTX.

This observation indicates that, compared to PGE_2_, MTX exhibits greater local flexibility during its binding process with hABCC4. It also implies that hABCC4 exhibits varying stringency and flexibility when binding different ligands, and that some flexibility may be involved in the binding process of hABCC4 with cAMP.

However, to confirm the flexible binding of MTX by hABCC4, an issue remains to be addressed: the MTX ligand exhibits poor density in the W995-R998 region in both the 9KRM and 8BWP structures. Based solely on the structural model data we obtained, it cannot be definitively proven that the sparse density in this region belongs to the MTX ligand. To resolve this issue, we designed a cellular MTX transport assay to confirm the critical roles of R946, Q994, and R998 in MTX binding. This assay would help determine whether the density near the W995-R998 region in the protein model corresponds to MTX.

**Fig. 5:**
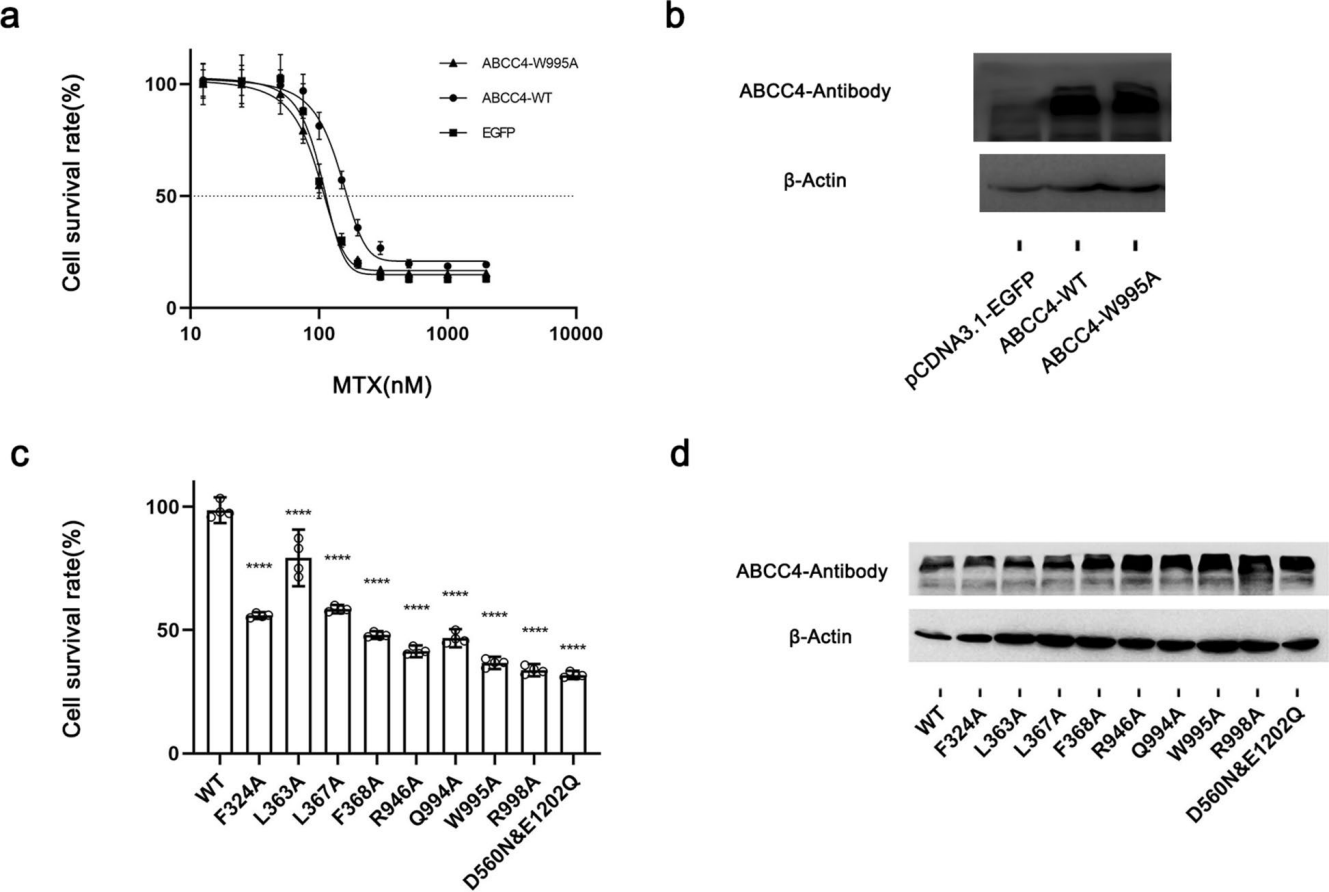
Impact of hABCC4 Residue Mutations on MTX Transport. To confirm whether the poorly distributed density in the W995-R998 region of the binding pocket in the 9KRM structure corresponds to the MTX ligand, we designed transport assays to evaluate the impact of the relevant residues on MTX binding. **a** Activity of HEK293T cells transfected with ABCC4-WT, ABCC4-W995A, or pCDNA3.1-EGFP after exposure to different concentrations of MTX for 48 h (data are presented as the means ± SEMs). This result revealed that the optimal MTX concentration for residue mutation transport experiments is 100 nmol/L. **b** Western blot results showing hABCC4 protein expression in HEK293T cells transfected with ABCC4-WT, ABCC4-W995A, or pcDNA3.1-EGFP. **c** Activity of HEK293T cells transfected with hABCC4 mutant vectors in the presence of 100 nM MTX (data are presented as the means ± SEMs). **d** Western blotting results demonstrating protein expression in HEK293T cells transfected with mutant hABCC4 vectors

The transport of MTX by hABCC4 was assessed via the Cell Counting Kit (CCK)-8 reagent. In a preliminary experiment, we assessed the activity levels of HEK293T cells transfected with hABCC4-WT or W995A fused with an EGFP tag. As a control, cells were transfected with the EGFP expression vector, and their activity levels were assessed at various concentrations of MTX. The experimental findings revealed that the EC_50_ values for cells transfected with WT, W995A, and the control group (EGFP) were 151.9, 80.21, and 79.32 nmol/L, respectively (Fig. 5a). To compare the EC_50_ results between hABCC4-WT and control cells, subsequent experiments were conducted with a MTX concentration of 100 nmol/L.

In a formal experiment, cells expressing different EGFP mutant hABCC4 fusion vectors were exposed to MTX medium at a concentration of 100 nmol/L, as depicted in Fig. 5c. Single point mutations associated with MTX binding exert varying degrees of influence on its transport, with R946, Q994, W995, and R998 mutations having the most significant impact on helix TM12. The obtained results aligned with the anticipated impact of the residues in our structural model. This result demonstrates that the density near the W995-R998 region corresponds to MTX and confirms the flexibility of hABCC4 in binding to MTX.

A comparison of our 9KRM and 9KRN models with the 8BWP and 8BWR models demonstrated that the structural models we obtained, including 9KRK and 9KRL, as well as the cryo-EM sample preparation method used, are reliable. Furthermore, the comparison results suggest that hABCC4 is flexible in terms of substrate ligand recognition, and this binding flexibility varies between different substrates. This variability may also explain the suboptimal fit between the cAMP ligand density and the model in 9KRL.

### Exploration of a fluorescently labelled cAMP cellular transport assay

#### The impact of fluorescent moiety on the transport of 8-[Fluo]-cAMP

**Fig. 6:**
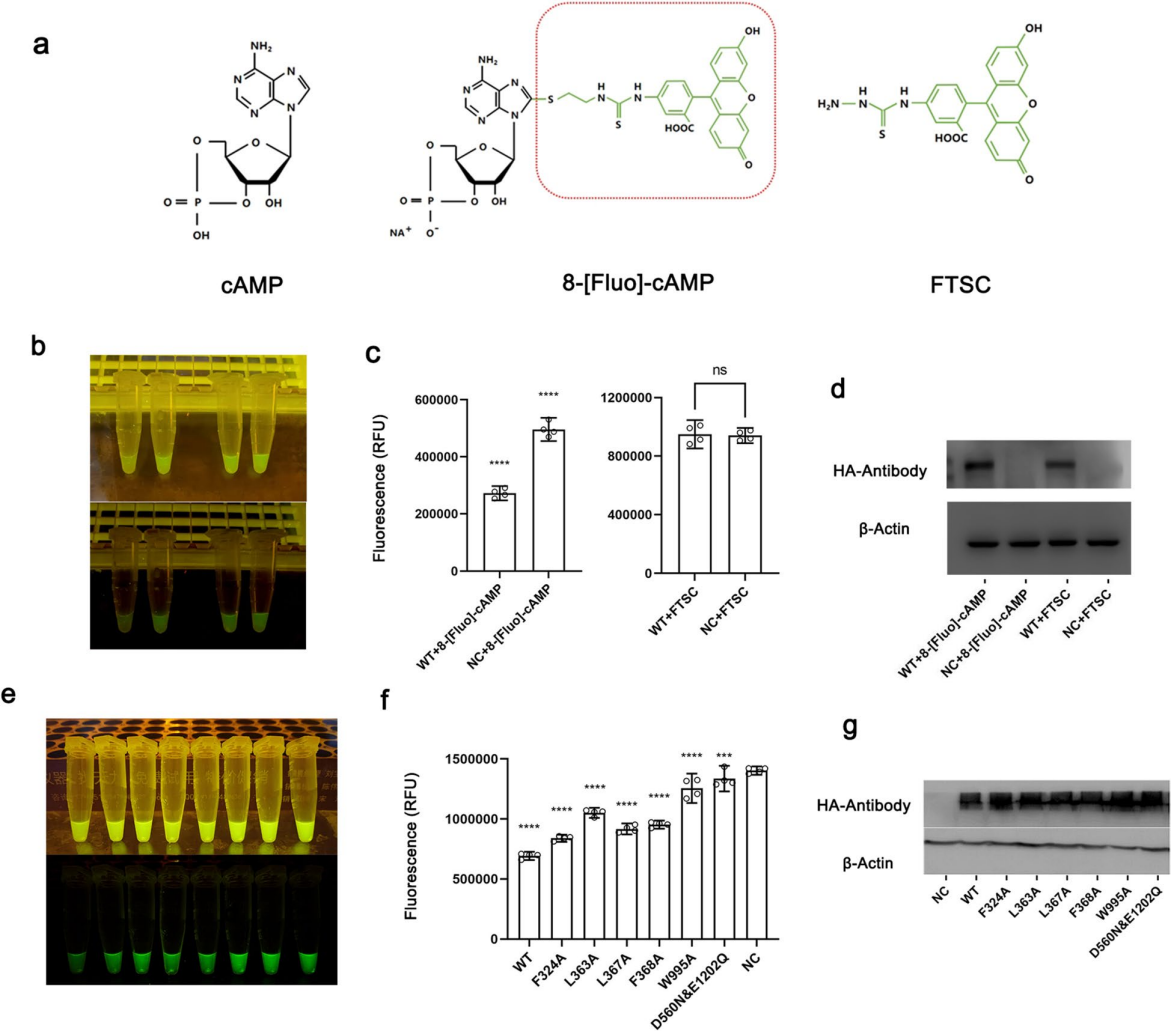
Assay of the efflux capacity of 8-[Fluo]-cAMP in HEK293T cells expressing hABCC4. To investigate the binding flexibility and tolerance of hABCC4 toward cAMP, as well as the potential of 8-[Fluo]-cAMP as a detection tool in transport assays, we designed cellular transport experiments to examine the impact of key residue mutations involved in hABCC4 recognition and binding of cAMP on the transport of 8-[Fluo]-cAMP. **a** The molecular structures of cAMP, 8-[Fluo]-cAMP, and FTSC are presented. The fluorescent tag regions, which may influence hABCC4 recognition, are highlighted by red dashed lines. **b** After incubation with 8-[Fluo]-cAMP and FTSC, cell lysate supernatants from cells expressing hABCC4-WT or the empty vector pCDNA3.1 are shown. Each sample shown in the figure was randomly selected from four parallel samples in the corresponding experimental group. The upper panel shows the original images, whereas the lower panel shows images with adjusted brightness to enhance the visibility of green fluorescence. **c** Fluorescence intensity of the cell lysates from (**b**) was measured via a fluorescence microplate reader (n = 4; data are presented as the means ± SEMs). The results demonstrated that cells transfected with hABCC4-WT exhibited significant 8-[Fluo]-cAMP efflux activity, whereas no significant transport activity was observed for FTSCs. **d** Western blot analysis (WB) of hABCC4 protein expression in cell lysates from the 8-[Fluo]-cAMP and FTSC transport experiments. **e** Cell lysates from HEK293T cells expressing various hABCC4 residue mutants after incubation with 8-[Fluo]-cAMP. **f** Fluorescence intensity of the cell lysates from (**e**) was measured via a fluorescence microplate reader (n = 4; data are presented as the means ± SEMs). **g** Western blot analysis (WB) of hABCC4 protein expression in cell lysates from cells transfected with various hABCC4 residue mutants

To utilize 8-[Fluo]-cAMP as a tool for detecting cAMP transport, a prerequisite issue must be addressed: the potential impact of the fluorescent labelling moiety on 8-[Fluo]-cAMP. We selected fluorescein-5-thiosemicarbazide (FTSC, Fig. 6a), a fluorescent molecule structurally similar to the fluorescent moiety of 8-[Fluo]-cAMP, as a control for the cellular transport assay. If cells expressing hABCC4 can transport 8-[Fluo]-cAMP but not FTSC, this would indirectly demonstrate that the cAMP portion of the 8-[Fluo]-cAMP molecule is responsible for hABCC4-mediated transport.

We modified the 8-[Fluo]-cAMP cellular transport assay method on the basis of previously published studies [42]. HEK293T cells transfected with a pCDNA3.1 plasmid encoding hABCC4 with an N-terminal HA tag were used for the transport assay. After 48 h of transfection with either hABCC4-WT or the empty vector pCDNA3.1 (NC), the cells were incubated with 8-[Fluo]-cAMP or FTSC. The fluorescence intensity of the residual intracellular fluorescent molecules was then measured via a fluorometer, with both 8-[Fluo]-cAMP and FTSC detected at an excitation wavelength of 494 nm and an emission wavelength of 517 nm.

The results of the 8-[Fluo]-cAMP assay revealed that the residual fluorescence in cells transfected with ABCC4-WT was significantly lower than that in cells transfected with the NC plasmid (Fig. 6b). In contrast, in the FTSC assay, the residual fluorescence was comparable between the ABCC4-WT and NC groups (Fig. 6c). This finding indicates that, under our experimental conditions, FTSC is not transported by hABCC4, whereas 8-[Fluo]-cAMP is. These findings suggest that the fluorescent labelling moiety of the 8-[Fluo]-cAMP molecule has a negligible effect on its transport by hABCC4.

#### Mutations in residues of hABCC4 affect the transport of 8-[Fluo]-cAMP

The FTSC transport assay demonstrated that hABCC4 recognizes the cAMP moiety of 8-[Fluo]-cAMP during transport. Combined with the structural information obtained from the Cryo-EM analysis of the complex, these findings suggest that hABCC4 exhibits considerable flexibility in cAMP binding. This flexibility may provide recognition tolerance and spatial flexibility, enabling hABCC4 to transport 8-[Fluo]-cAMP similarly to cAMP, despite its larger fluorescent tag. To validate this hypothesis, we investigated whether hABCC4 consistently recognizes cAMP and 8-[Fluo]-cAMP using transport functional assays. We constructed a series of expression vectors encoding HA-tagged hABCC4 mutants, with mutations identified from the hABCC4-cAMP complex structure. These vectors were used to transfect HEK293T cells, and changes in 8-[Fluo]-cAMP transport capacity were evaluated.

hABCC4-WT and all the mutants were transfected into HEK293T cells, and fluorescence was directly observed under a microscope after treatment with 8-[Fluo]-cAMP (Fig. 6e). The fluorescence intensity of the WT was significantly lower than that of the other mutants (Fig. S1). The cell samples were then collected and processed, and the fluorescence intensity was measured via a fluorometer. The calculated fluorescence values are shown in Fig. 6f. The results demonstrated that the W995 mutation had the most significant impact on cAMP transport, while mutations at F324, L363, L367, and F368 also affected transport to varying degrees, although none as prominently as W995. These results suggest that hABCC4 likely binds 8-[Fluo]-cAMP in a manner similar to its recognition and binding of cAMP. However, while these findings highlight the potential of 8-[Fluo]-cAMP as a tool for studying hABCC4-mediated cAMP transport, further critical validations are needed before its full application. This is further elaborated in the Discussion section.

## Discussion

### The substrate binding flexibility of hABCC4

ABCC4 is a functionally important transport protein in cells, and its functional diversity is reflected in its ability to recognize and transport a wide variety of substrates. This diversity requires ABCC4 to accurately recognize and bind these ligand molecules. However, according to existing structural information, the substrate-binding pocket of ABCC4 is a relatively fixed structural region. This suggests that the binding pocket may employ a certain degree of flexibility in its recognition strategy to accommodate different substrate ligands. Clues to this strategy can be found in the structures of hABCC4 bound to MTX and PGE_2_ that we obtained.

The published structure 8BWP (Map: EMDB ID EMD-16293) [45] and the map of the structure we obtained, 9KRM, reveal a notable phenomenon in the density distribution of the MTX ligand molecule. Specifically, regions of high local density are interspersed with areas of low density, discontinuity or absence. The differences between the two models suggest that the binding of MTX within the hABCC4 pocket is flexible. MTX binding near residues F324, L367, F368, and W995 is shown to be tight and precise, as both models clearly show density in this region. However, in areas outside this region of the pocket, particularly near residues R946, Q994, and R998, MTX binding is relaxed, resulting in sparse density. These residues, however, still play a significant role in MTX binding. MTX, a widely used anticancer drug, is a derivative of folate, a natural substrate transported by ABCC4 [46]. Although current evidence is insufficient to conclude that ABCC4 transports MTX due to its misrecognition as folate, the flexible recognition mechanism of ABCC4 may explain why this synthetic molecule, which does not naturally occur in biological systems, can also be transported. This phenomenon of flexible binding may be a key factor underlying the substrate diversity in ABCC4 ligand recognition.

In the map of our obtained structure 9KRN, the density of the PGE_2_ ligand is well defined and fits the molecular model accurately, without the coexistence of high- and low-density regions observed in the MTX ligand map of 9KRM. This finding indicates that the binding flexibility of hABCC4 for PGE_2_ is significantly lower than that for MTX, indicating that ABCC4 exhibits varying degrees of flexibility tolerance depending on the substrate ligand.

The suboptimal fit of the cAMP ligand model in our obtained 9KRL structure may also stem from the flexible recognition characteristics of ABCC4. On the basis of the map we obtained, the density of the cAMP ligand is almost entirely concentrated near the W995 residue. Although residues such as F324, L363, L367, and F368 are also located near the density of cAMP on the basis of their side chains, these hydrophobic residues interact weakly with the hydrophilic cAMP molecule. The π‒π stacking interactions with W995 are unique and critical for cAMP binding, whereas residues F324, L363, L367, and F368 provide relatively weaker stabilizing effects.

**Fig. 7:**
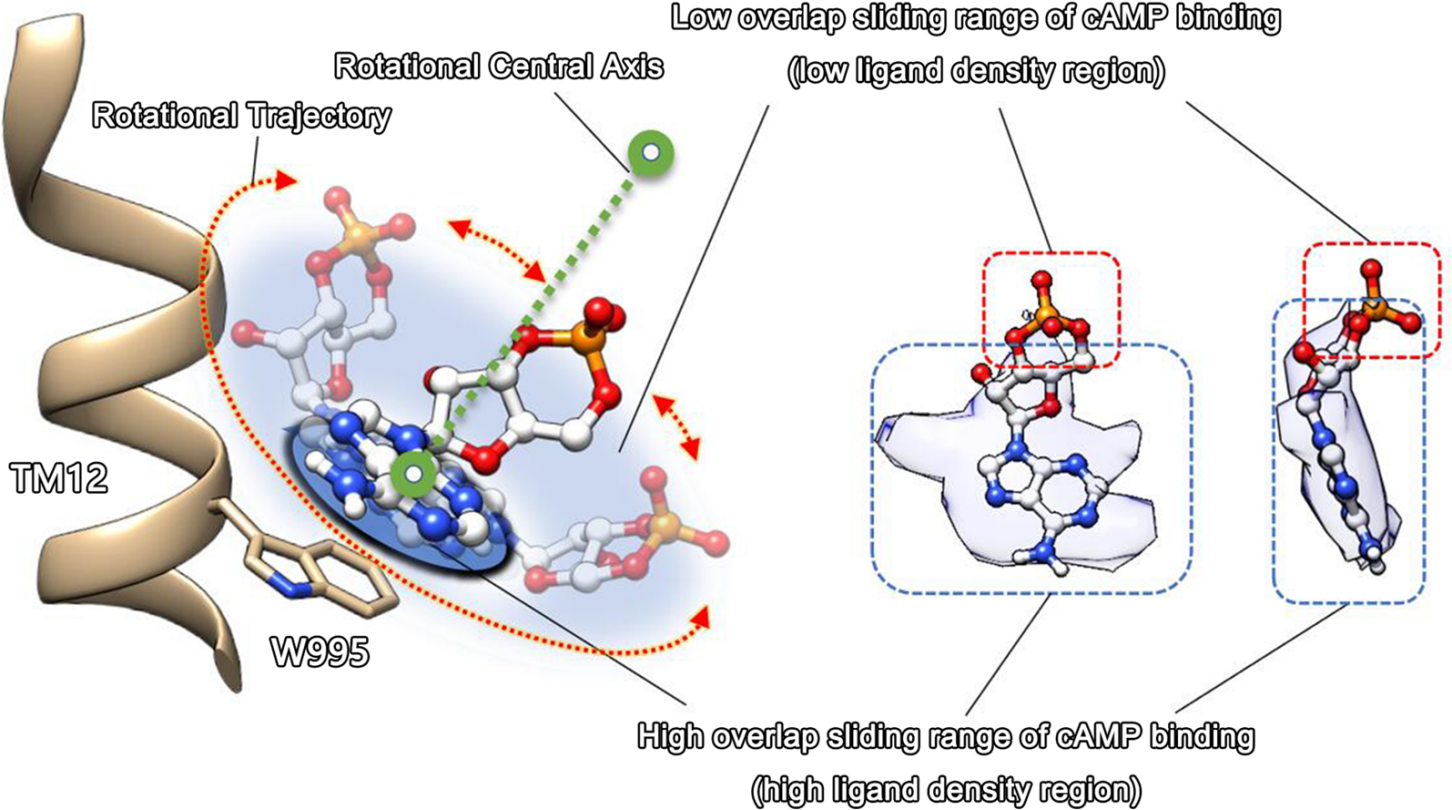
Flexible binding of cAMP to hABCC4. Information obtained from cryo-EM structural data revealed that the W995 residue plays a critical role in the binding of cAMP to hABCC4. Other potentially involved residues primarily contribute through hydrophobic interactions. However, these interactions are likely to have minimal effects on the binding of the hydrophilic cAMP molecule. As a result, cAMP is attracted and bound by W995 within the binding pocket. Owing to the lack of additional strong binding residues to stabilize it, the cAMP molecule undergoes flexible sliding. One of the rings of the cAMP molecule is closely aligned with the piperidine ring of W995 through π‒π stacking interactions. The remaining parts of the cAMP molecule undergo flexible sliding within a circular range centered around the piperidine ring. Regardless of the orientation in which cAMP is bound, the portion of the ligand near W995 consistently overlaps. This produces a disc-shaped, well-defined density, whereas the remaining parts fail to generate clear density

This may result in a specific binding mode with significant flexibility, allowing portions of the cAMP molecule outside the vicinity of W995 to adopt a highly flexible binding conformation. This flexibility likely leads to the inability to capture the density of certain regions of the cAMP ligand (as described in Fig. 7), ultimately resulting in a high overall resolution for the hABCC4 protein structure but severe density loss for the substrate ligand.

This hypothesis of flexible binding may help explain certain theoretical questions regarding the transport of 8-[Fluo]-cAMP by hABCC4. For example, this flexible binding mechanism likely allows hABCC4 to accommodate cAMP even when it carries a relatively large fluorescent tag. Furthermore, this characteristic of hABCC4 in substrate ligand recognition could provide valuable insights for molecular design and modification. For example, when new drugs are designed on the basis of their structure, targeted modifications could be made to the molecular regions that are tightly recognized and bound by hABCC4. This approach could lead to the development of novel anticancer drugs capable of evading ABCC4-mediated efflux.

In addition, a noteworthy issue is the potential relationship between the ligand-binding flexibility of hABCC4 and its stimulated ATPase activity. Among the ligands in the hABCC4 complex structures we obtained, PGE_2_ has the lowest binding flexibility, MTX shows higher flexibility than PGE_2_, and cAMP has the highest flexibility. Correspondingly, PGE_2_ strongly stimulates the ATPase activity of hABCC4, MTX weakly stimulates it, and cAMP does not stimulate ATPase activity in hABCC4 [33, 45].

In fact, a structural study on ABCC1 [47] revealed that when ABCC1 binds the substrate ligand LTC_4_, the LTC_4_ molecule serves as a bridging mediator in the substrate-binding pocket, bringing the two TMD domains closer. This induces a conformation that facilitates the alignment of the NBD domains, promoting NBD dimerization and significantly enhancing ATPase activity. This suggests that, in ABC family proteins, the bridging mediator role of substrate ligands is linked to stimulated ATPase activity. The higher the ligand's flexibility, the less stable its bridging mediator role may be, limiting the generation of stimulated ATPase activity.

Unfortunately, our study lacks sufficient evidence to determine if this explains why cAMP fails to stimulate ATPase activity in hABCC4. However, as a potential research direction, this warrants further investigation.

### Challenges to be addressed for the application of 8-[Fluo]-cAMP in cAMP transport assays

Compared with complex in vitro experiments that require the extraction of large amounts of membrane proteins, if 8-[Fluo]-cAMP can be utilized as a research tool to study the transport of cAMP by ABC proteins, it would significantly reduce the limitations associated with cAMP-related research methods and facilitate broader applications in this field. Our study highlights the characteristics of hABCC4 binding to cAMP and identifies key amino acids involved in this process. Furthermore, we demonstrated the potential recognition consistency between hABCC4-mediated transport of cAMP and 8-[Fluo]-cAMP by analysing transport changes in cells expressing mutant proteins. In addition, through FTSC transport experiments, we largely ruled out the potential impact of the fluorescent tag carried by 8-[Fluo]-cAMP. These findings suggest the potential feasibility of using 8-[Fluo]-cAMP as an experimental tool.

Some studies have hypothesized that the transport of cAMP by ABCC4 is not a direct process but rather a secondary phenomenon resulting from ABCC4-mediated prostaglandin transport. Therefore, in the design of the cell-based experiments, the final concentration of 8-[Fluo]-cAMP added to the extracellular supernatant of HEK293T cells overexpressing hABCC4 was set to 0.02 mM. At this external concentration, the intracellular uptake of 8-[Fluo]-cAMP was significantly increased. Under these intracellular conditions, hABCC4, when strongly expressed, is the only transporter capable of significantly reducing intracellular 8-[Fluo]-cAMP levels. In cells with high hABCC4 expression, the effects of various prostaglandins on intracellular cAMP levels are diminished.

However, several unresolved issues remain in our study. First, although we confirmed that hABCC4 can bind cAMP by obtaining the hABCC4-cAMP complex structure and proposed insights into how hABCC4 recognizes and binds substrate ligands such as cAMP on the basis of structural information, owing to limitations in experimental facilities and resources, we were unable to further demonstrate whether hABCC4 directly transports cAMP. While the structural information provided predictions about the effects of specific residues on the transport of 8-[Fluo]-cAMP, we were unable to experimentally confirm whether these residues similarly influence the transport of cAMP, as predicted. This requires better-equipped research teams to design more appropriate experimental approaches.

Additionally, although our cell-based experiments demonstrated an association between hABCC4 and the transport of 8-[Fluo]-cAMP, we acknowledge that more direct evidence is needed to confirm that hABCC4 directly recognizes, binds, and transports 8-[Fluo]-cAMP. Despite our findings that, if hABCC4 directly transports 8-[Fluo]-cAMP, it specifically recognizes and binds the cAMP portion of the molecule, further validation of the direct transport relationship between hABCC4 and 8-[Fluo]-cAMP requires additional experiments. These methods could include reconstituted protein vesicle transport assays, radiolabelled molecular tracing, or obtaining the protein complex structure of ABCC4 bound to 8-[Fluo]-cAMP.

### Methods

#### Expression and purification of hABCC4

The hABCC4 gene (UniProt ID: O15439) was synthesized and ligated into the pFastbac1 plasmid. It was incorporated into an hABCC4 fusion protein expression vector with a dual tag consisting of an 8 × His tag and a maltose-binding protein (MBP) at the N-terminus. The hABCC4 gene was subjected to point mutation treatment, and a mutant hABCC4 vector was constructed via the same methodology.

The hABCC4 protein was expressed via baculovirus generated from SF9 cells via the Bac-to-Bac system (Invitrogen). SF9 cells were cultured in suspension at a density of approximately 5 × 105/mL and subsequently infected with P2 virus at a ratio of 1:20 (v:v). After 48 h, the cells were harvested and resuspended in Buffer A (pH 7.8, 50 mM Tris–HCl, 5% glycerol, and 500 mM NaCl). The suspension was supplemented with protease inhibitors (1%, v/v; Roche), followed by disruption via a high-pressure homogenizer. The lysate was centrifuged at 42,000 rpm and 4 °C for 1 h. The collected cell membranes were homogenized in buffer A. The obtained homogenates were subsequently incubated at 4 °C for 2 h with the detergents LMNG (1%, v/v, Anatrace), CHS (0.2%, v/v, Anatrace), and protease inhibitors. After incubation, the supernatant was collected by centrifugation at 8000 rpm and 4 °C for 1 h. Subsequently, Ni affinity resin (New England Biolabs) was added and allowed to bind at 4 °C for 2 h. The Ni resin was collected by centrifugation at 2000 rpm for 2 min. After the resin was cleaned with buffer B (20 mM imidazole, 0.005% LMNG, and 0.001% CHS were added to buffer A), the hABCC4 fusion protein was eluted with buffer C (500 mM imidazole, 0.005% LMNG, and 0.001% CHS were added to buffer A). The hABCC4 fusion protein obtained by elution from the Ni resin was subsequently bound to the MBP affinity resin (New England Biolabs) for 1 h, followed by collection of the resin by centrifugation. After the resin of the MBP was cleaned with Buffer C (0.005% LMNG and 0.001% CHS were added to Buffer A), the hABCC4 fusion protein was eluted with Buffer D (20 mM maltose, 0.005% LMNG, and 0.001% CHS were added to Buffer A).

The hABCC4 fusion protein was concentrated to approximately 6–8 mg/mL via a 100 kDa protein concentrator (Millipore). The peak fraction was then collected on a Superose 6 chromatographic column (GE Healthcare) for further concentration and was subsequently used in the preparation of cryo-EM samples.

### ATPase assay

The ATPase activity of hABCC4 was calculated on the basis of the amount of phosphate generated from ATP hydrolysis. The hABCC4 solution (the concentration of hABCC4 protein was 0.94 mg/mL) was mixed and preincubated at 4 °C in low salt buffer, along with solutions containing varying concentrations of ATP and MgCl_2_ (molar ratio of ATP to Mg^2+^ 1:2, Sigma‒Aldrich). The resulting mixtures were combined in equal volumes to initiate hydrolysis. The mixture was incubated at 37 °C for 15 min and subsequently terminated by rapid freezing in liquid nitrogen. A phosphate assay kit (Sigma‒Aldrich) was used to measure the amount of phosphate produced during the hydrolysis reaction, following the instructions provided.

The ATPase activity of hABCC4 was assessed in the presence of varying concentrations of PGE_2_ (ranging from low to high) while maintaining a constant ATP concentration of 4 mM to quantify the extent of stimulation caused by PGE_2_. An appropriate concentration of PGE_2_ (MedChemExpress) was prepared, dissolved in double-distilled water and added to the experimental system. The experimental method employed was consistent with the previous approach used for detecting the basal ATPase activity of hABCC4 while reducing the reaction time from 15 to 5 min at 37 °C. The data were subsequently analysed and fitted via GraphPad Prism 9.3.

### Transport capacity assay

#### cAMP

The pcDNA3.1 plasmid vectors containing the hABCC4 gene, including the WT, F324A, L363A, L367A, F368A, W995A, D560N, and E1202Q variants, were constructed. Vectors containing HA tags at the N-terminus of hABCC4.

HEK293T cells were seeded in six-well plates at a density of 5 × 10^5^ cells/well. After incubation for 4–5 h to allow cell attachment, Lip8000 transfection reagent (Beyotime Biotechnology, Shanghai) was used to transfect the hABCC4 plasmid and pcDNA3.1 empty vector (NC) into HEK293T cells. Each plasmid was transfected into three parallel six-well plates. After 48 h of transfection, the cells from three parallel wells in each plasmid transfection group were resuspended, collected, evenly mixed, and divided into four equal portions. After centrifugation, the supernatant was discarded, and complete DMEM (10% fetal bovine serum [FBS]) containing 8-[Fluo]-cAMP (BioLog Life Science Institute) or FTSC (Macklin, China) was added. The concentration of 8-[Fluo]-cAMP or FTSC was 0.02 mM. The mixture was allowed to incubate for 1 h. Following incubation, the culture medium was subjected to centrifugation. After cell counting, the number of cells used in all the experimental groups was consistent. The cells obtained through centrifugation were then collected. The cells were rinsed twice with phosphate-buffered saline (PBS). The PBS supernatant was discarded after centrifugation. The cells were then lysed overnight with 100 µL of NP40 (Beyotime Biotechnology, Shanghai). After cell lysis, the lysate was centrifuged at 13,000 rpm for 10 min to remove cell debris, and the supernatant was collected for fluorescence intensity measurement. The fluorescence of the supernatant was first roughly observed and recorded under a fluorescent lamp, followed by precise quantification using a fluorescence spectrophotometer. Higher fluorescence values in the lysate supernatant indicate a greater amount of fluorescently labeled molecules retained within the cells, reflecting weaker protein transport activity. The fluorescence intensity of the lysate supernatant was used to evaluate the impact of ABCC4 on the transport of 8-[Fluo]-cAMP or FTSC (the excitation and emission wavelengths for 8-[Fluo]-cAMP and FTSC are 494 nm and 517 nm, respectively).

A Western blot analysis was performed using an HA antibody (Proteintech, 51,064–2-AP) and a β-actin antibody (Proteintech, 81,115–1-RR). The secondary antibodies used were goat anti-rabbit antibody (Proteintech, RGAR001) and goat anti-rat antibody (Cohesion Biosciences, CSA2162). The plasmid was transfected into HEK293T cells under the same conditions as in the transport experiment and then incubated for 48 h. Subsequently, the cells were lysed in RIPA lysis buffer (MedChemExpress) on ice for 30 min. After 10 min of sonication, 5 × loading buffer (Beyotime Biotechnology, Shanghai) was added, and the mixture was heated at 100 °C for 10 min. The resulting supernatant, obtained after the removal of cell fragments by centrifugation, was used for SDS‒polyacrylamide gel electrophoresis (PAGE). After electrophoresis via a 12% SDS‒PAGE gel, the protein samples were transferred to a PVDF membrane (0.22 μm, Beyotime Biotechnology, Shanghai) at 230 mA on ice for 2 h and 10 min. The PVDF membrane was initially blocked for 1 h at 20 °C. The samples were then incubated overnight at 4 °C in primary antibody solution. The following day, the PVDF membrane was washed with PBST and incubated with a secondary antibody solution for 1 h. Subsequently, the membrane was rinsed with PBST and treated with an enhanced chemiluminescence (ECL) solution (Yamei, Shanghai) to capture images.

#### MTX

The pcDNA3.1 plasmid vectors containing the hABCC4 gene, including WT, F324A, L363A, L367A, F368A, Q994A, W995A, R998A, D560N, and E1202Q, were constructed. Vectors containing EGFP tags at the N-terminus of hABCC4.

HEK293T cells were seeded in 24-well plates at a density of 10 × 10^4^ cells/well and allowed to adhere for 4 h. The hABCC4 plasmid and pcDNA3.1 empty vector (NC) were separately transfected into four sets of parallel wells containing HEK293T cells for 24 h.

MTX (0.908 g; MedChemExpress) was dissolved in DMSO (1 mL). Subsequently, 10 µL of this mixture was transferred to 1 mL of DMEM (Solarbio, Beijing), and complete dissolution in complete medium containing 10% FBS was ensured. Finally, different concentrations were prepared by further dilution according to the experimental requirements.

After 24 h of transfection, the cell supernatant was discarded, and DMEM supplemented with varying concentrations of MTX was added for incubation. After 48 h of reculture, the DMEM containing MTX was discarded, and the medium was replaced with DMEM supplemented with CCK-8 reagent (Cell Counting Kit-8, MedChemExpress). After 3 h of incubation for color development, the reaction was terminated, and samples were collected for colorimetric analysis via an enzyme-linked microplate reader. The wavelength was set at 450 nm, and cell viability was calculated on the basis of the manufacturer’s instructions.

Western blotting was performed using an ABCC4 antibody (Abcam, ab15602) and a β-actin antibody (Proteintech, 81,115–1-RR). The secondary antibody used was goat anti-rat IgG (H&L)-HRP (Cohesion Biosciences, CSA2162). The parameters used for western blotting were in accordance with those used in a previous cAMP experiment.

#### Cryo-EM sample preparation and data acquisition

Purified hMRP4 was concentrated to a concentration of 6–8 mg/mL in a 100 kDa protein concentration tube (Millipore). cAMP (MedChemExpress) and PGE_2_ (MedChemExpress) were dissolved in water, whereas MTX (MedChemExpress) was dissolved in DMSO. Substrates were added to the purified protein mixture at a concentration of 10% of the total volume of the hABCC4 protein mixture. The final concentrations of the cAMP, MTX, and PGE_2_ substrates were 7.29 mM, 0.11 mM, and 5.66 mM, respectively, for a final ATP concentration of 10 mM. The samples were combined and allowed to sit for 30 min prior to being prepared for cryo-EM. Using a Vitrobot Mark (Thermo Fisher Scientific), the samples were prepared after the carrier mesh (300-mesh copper grid, Quantifoil) received a hydrophilic treatment at 0.26 atmosphere and 25 mA for 45 s. Vitrobot conditions were set to 4 °C and 100% humidity. The protein sample (5 µL) was added to the grid. After the excess protein sample was removed via filter paper, the grid was rapidly frozen in liquid ethane. The prepared carrier mesh samples were stored in liquid nitrogen until further use.

Initial screening for cryo-EM was conducted using an FEI 200 kV Arctica transmission electron microscope (TEM). Mesh samples with an optimal particle density and moderate ice thickness were carefully selected for subsequent data collection. After the grids were selected, the samples were loaded into an FEI 300 kV Titan Krios electron microscope for high-resolution protein structure data collection. The data collection was assisted by a Gatan K2 electron detector, a Gatan quantum energy filter (20 eV), and a spherical aberration corrector. The magnification for the protein particles was set at 64,000x, corresponding to a pixel size of 0.55 Å on the image. The exposure time was set to 7.2 s, with an average dose of 1.5 e/Å^2^ per micrograph, totaling 40 frames and an accumulated dose of approximately 60 e/Å^2^. Data were collected via the FEI EPU program, and the nominal defocus value ranged from − 1.2 to − 2.2 µm.

#### Cryo-EM data processing

The movie stacks were processed via MotionCor2 [48] for beam-induced motion correction and dose weighting to rectify the single-pixel horizontal anisotropic motion across the entire frame. This was followed by additional correction via RELION 3.0 [49] (pixel size of 1.1 Å). Contrast transfer function (CTF) parameters were determined through patch-based CTF analysis in cryoSPARC [50]. Protein particle picking and 3D classification were conducted via cryoSPARC’s ‘blob-picker. Through multiple cycles of two-dimensional classification, the influences of ice particles, false-positive particles, and damaged protein particles were eliminated. An optimization stage was initiated for the selected particles, with a focus on those exhibiting a positive configuration difference, which may have arisen owing to the conformational flexibility of the protein. This process culminates in the reconstruction of the model (Figs. S1–S4).

#### Model building and structure refinement

To construct the hABCC4 protein structure model, we initially consulted the SWISS-MODEL database and established a preliminary model based on AlphaFold's predicted hABCC4 structure. Manual adjustments were subsequently made via UCSF Chimera to obtain further structural models, which were then compared with the clear structural positions identified in the density map obtained via cryo-electron microscopy. The resulting models underwent additional manual refinement with Coot [51] and were fine-tuned against the cryo-EM maps via Phenix [52]. The model quality was assessed with MolProbity [53]. Figures were created via PyMOL [54] and UCSF Chimera [55]. The final refinement statistics are listed in Table 1.

**Table 1:** Cryo-EM statistics for data collection, image processing, and model refinement

Sample	hABCC4-Apo	cAMP	MTX	PGE_2_
PDB code	9KRK	9KRL	9KRM	9KRN
EMDB code	EMD-62531	EMD-62532	EMD-62533	EMD-62534
Magnification	64,000	64,000	64,000	64,000
Voltage (kV)	300	300	300	300
Electron exposure (e–/Å^2^)	60	60	60	60
Defocus range (μm)	− 1.2 to − 2.2	− 1.2 to − 2.2	− 1.2 to − 2.2	− 1.2 to − 2.2
Pixel size (Å)	0.55	0.55	0.55	0.55
Initial particle images (no.)	2,207,375	3,118,394	3,639,983	3,639,700
Final particle images (no.)	111,741	383,488	358,889	350,509
Map resolution (Å)	3.29	2.99	3.00	3.14
FSC threshold	0.143	0.143	0.143	0.143
Map sharpening B factor (Å^2^)	− 129.5	− 136.3	− 138.3	− 150.8
Refinement				
Model resolution (Å)	3.6	3.3	3.2	3.4
FSC threshold	0.5	0.5	0.5	0.5
Model composition				
Nonhydrogen atoms	7575	7534	7641	7633
Protein residues	943	935	947	947
Ligands	0	1	1	1
R.m.s. deviations				
Bond lengths (Å)	0.002	0.004	0.002	0.004
Bond angles (°)	0.496	0.566	0.574	0.565
Validation				
MolProbity score	1.49	1.48	1.60	1.61
Clashscore	4.82	3.28	4.40	4.34
Poor rotamers (%)	0	0.2	0	0.1
Ramachandran plot				
Favored (%)	96.36	94.7	94.37	94.05
Allowed (%)	3.64	5.3	5.63	5.95
Disallowed (%)	0	0	0	0

## Supplementary Information

Additional file 1: Fig. S1 Fluorescence of HEK293T cells transfected with various ABCC4 mutants observed under a fluorescence microscope after treatment with 8-[Fluo]-cAMP. HEK293T cells were transfected with various hABCC4 expression vectors for 48 hours and then incubated in medium containing 8-[Fluo]-cAMP. Observations were made under a fluorescence microscope. In the left column (a), bright field images were captured, and in the right column (b), corresponding dark field images were captured (λ_exc_ 494 nm, λ_em_ 517 nm).

Additional file 2: Fig. S2 Cryo-EM Data Collection and Processing of hABCC4 (Apo). (a) Motion-corrected electron microscopy images. (b) Processing workflow of EM particle data. (c) Representative 2D classification of protein particle processing. (d) FSC data of the protein structure model reconstruction. (e) Resolution distribution of the density cloud. (f) Angular orientation results of the protein structure reconstruction.

Additional file 3: Fig. S3 Cryo-EM Data Collection and Processing of hABCC4 (cAMP-Bound). (a) Motion-corrected electron microscopy images. (b) Processing workflow of EM particle data. (c) Representative 2D classification of protein particle processing. (d) FSC data of the protein structure model reconstruction. (e) Resolution distribution of the density cloud. (f) Angular orientation results of the protein structure reconstruction.

Additional file 4: Fig. S4 Cryo-EM Data Collection and Processing of hABCC4 (MTX-Bound). Motion-corrected electron microscopy images. (b) Processing workflow of EM particle data. (c) Representative 2D classification of protein particle processing. (d) FSC data of the protein structure model reconstruction. (e) Resolution distribution of the density cloud. (f) Angular orientation results of the protein structure reconstruction.

Additional file 5: Fig. S5. Cryo-EM Data Collection and Processing of hABCC4 (PGE_2_-Bound). Motion-corrected electron microscopy images. (b) Processing workflow of EM particle data. (c) Representative 2D classification of protein particle processing. (d) FSC data of the protein structure model reconstruction. (e) Resolution distribution of the density cloud. (f) Angular orientation results of the protein structure reconstruction.

## Data Availability

The four hABCC4 structural models of Apo (9KRK), cAMP (9KRL), MTX (9KRM), and PGE_2_ (9KRN) have been uploaded to the Protein Data Bank (PDB). The cryo-EM density maps of the corresponding structures were deposited in the Electron Microscopy Data Bank (EMDB), ID: Apo EMD-62531, cAMP EMD-62532, MTX EMD-62533, PGE_2_ EMD-62534.
