# Supplementary figures and images for: Structural basis of human ABCC4 recognition of cAMP and ligand recognition flexibility
Source: Cell Biosci. 2025 Mar 27;15:39. doi: 10.1186/s13578-025-01377-y (PMC11948813; doi:10.1186/s13578-025-01377-y)

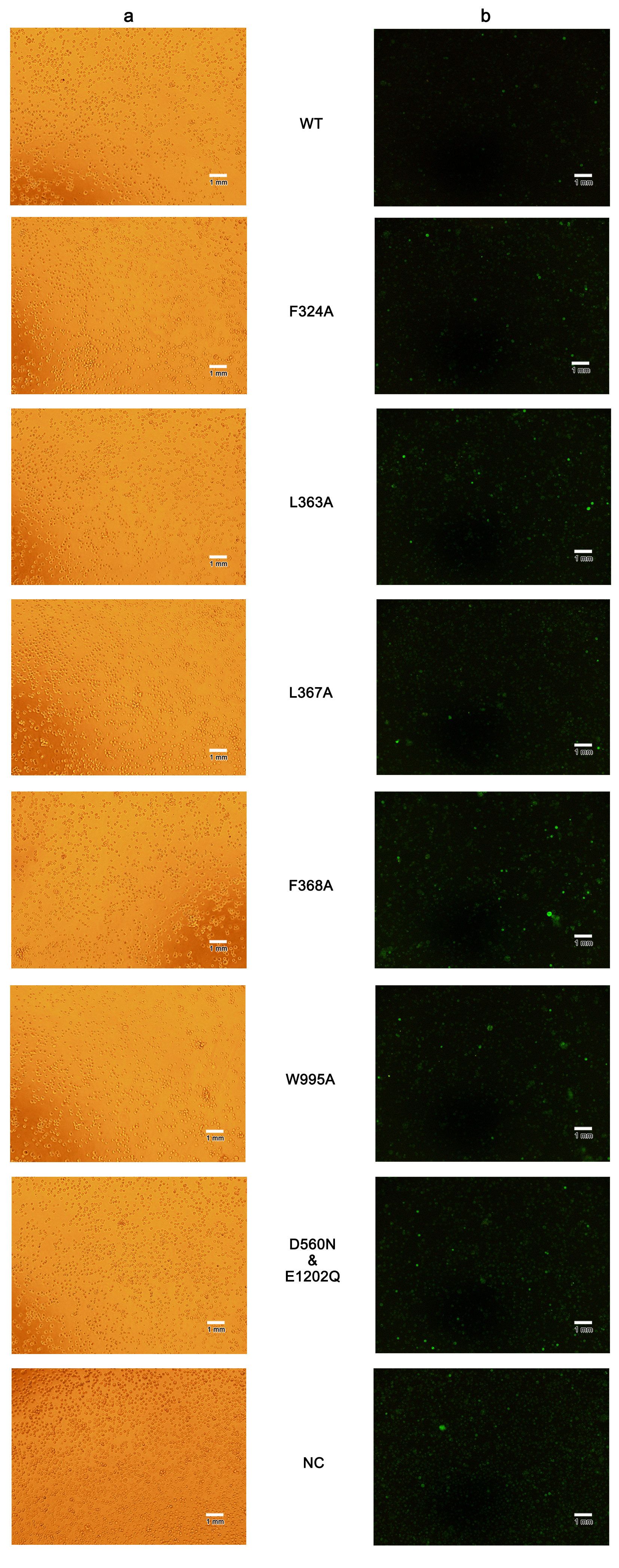

Supplement: Supplementary file 1 — Additional file 1: Fig. S1 Fluorescence of HEK293T cells transfected with various ABCC4 mutants observed under a fluorescence microscope after treatment with 8-[Fluo]-cAMP. HEK293T cells were transfected with various hABCC4 expression vectors for 48 hours and then incubated in medium containing 8-[Fluo]-cAMP. Observations were made under a fluorescence microscope. In the left column (a), bright field images were captured, and in the right column (b), corresponding dark field images were captured (λexc 494 nm, λem 517 nm). [file 13578_2025_1377_MOESM1_ESM.jpg]

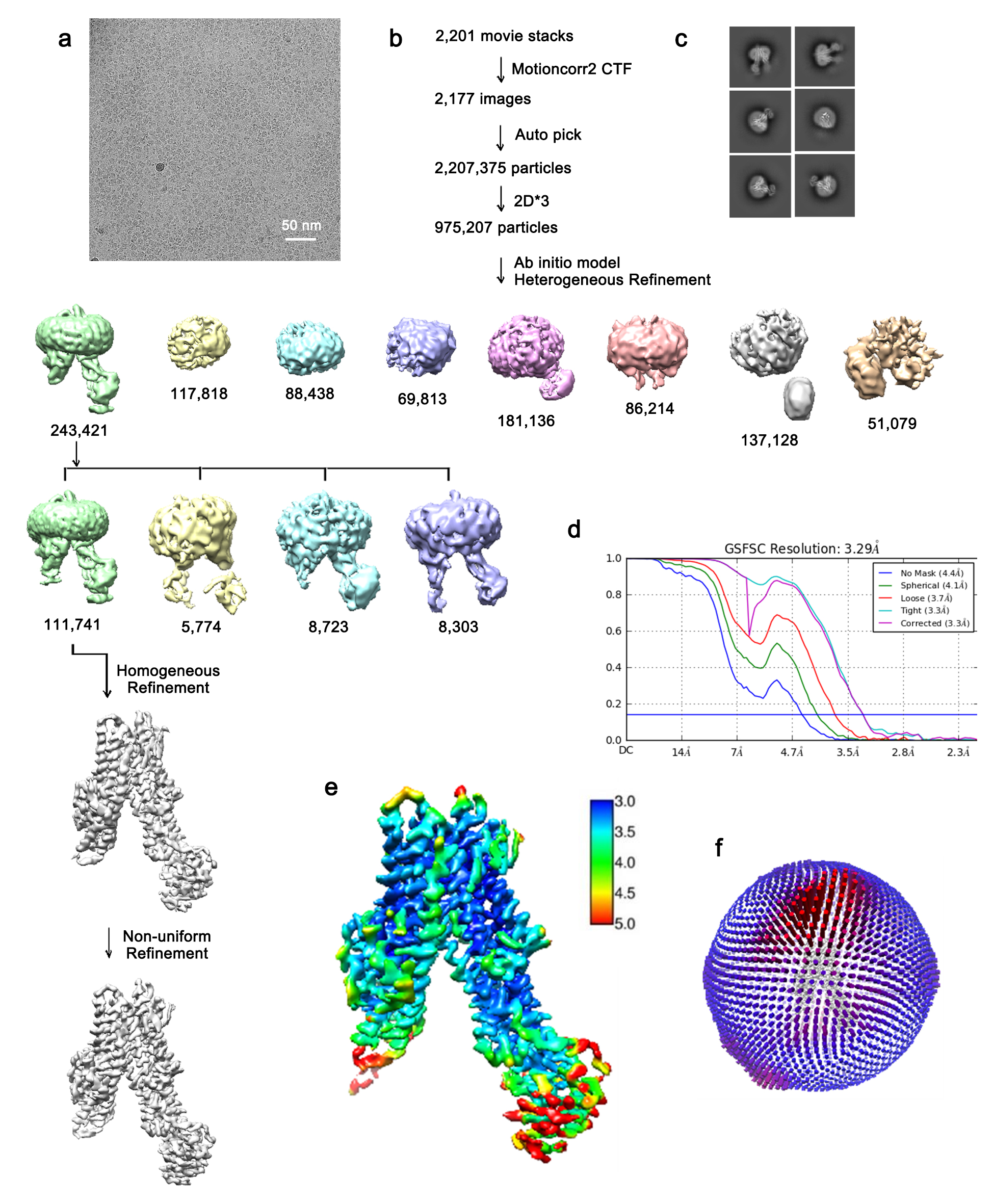

Supplement: Supplementary file 2 — Additional file 2: Fig. S2 Cryo-EM Data Collection and Processing of hABCC4 (Apo). (a) Motion-corrected electron microscopy images. (b) Processing workflow of EM particle data. (c) Representative 2D classification of protein particle processing. (d) FSC data of the protein structure model reconstruction. (e) Resolution distribution of the density cloud. (f) Angular orientation results of the protein structure reconstruction. [file 13578_2025_1377_MOESM2_ESM.jpg]

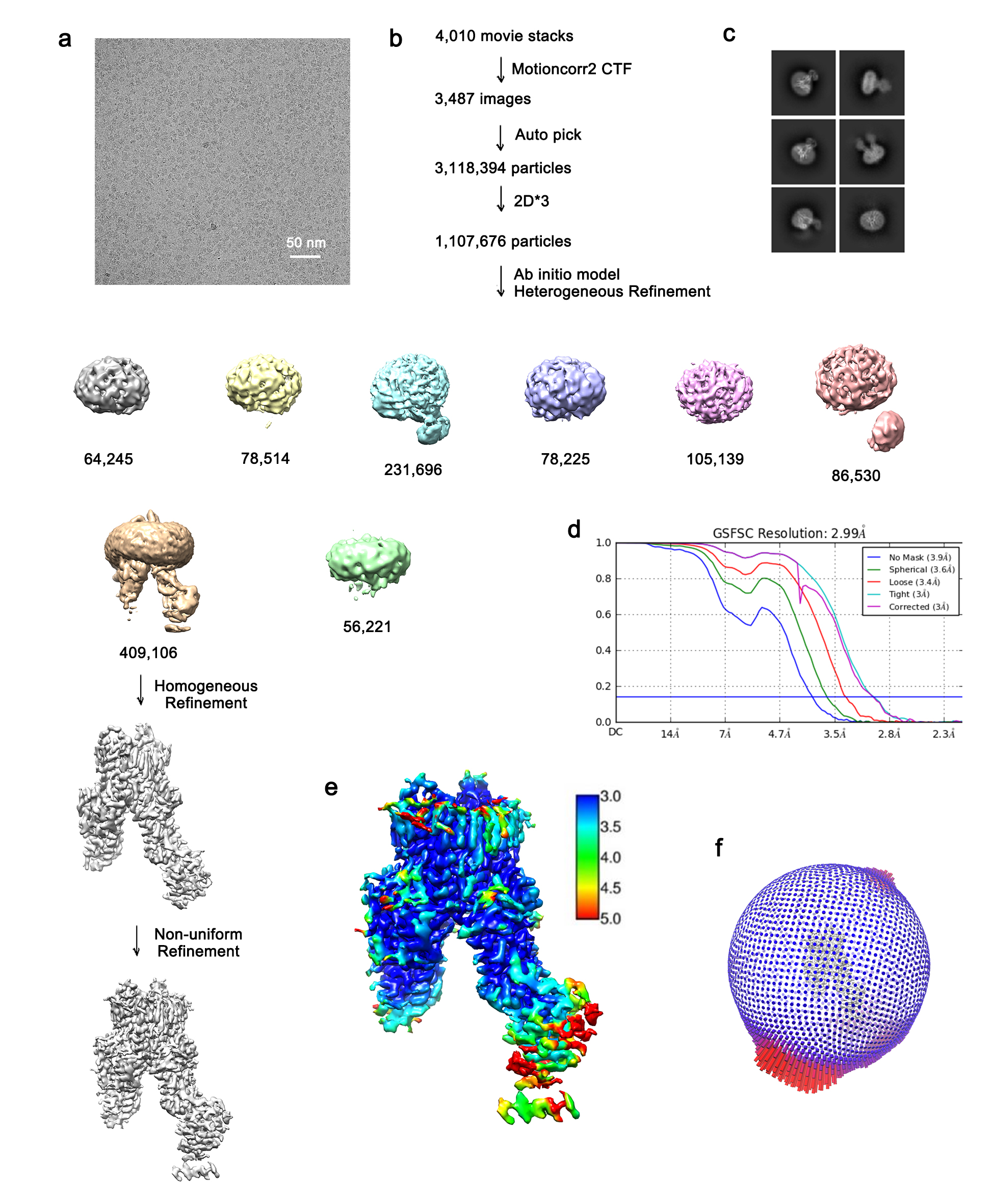

Supplement: Supplementary file 3 — Additional file 3: Fig. S3 Cryo-EM Data Collection and Processing of hABCC4 (cAMP-Bound). (a) Motion-corrected electron microscopy images. (b) Processing workflow of EM particle data. (c) Representative 2D classification of protein particle processing. (d) FSC data of the protein structure model reconstruction. (e) Resolution distribution of the density cloud. (f) Angular orientation results of the protein structure reconstruction. [file 13578_2025_1377_MOESM3_ESM.jpg]

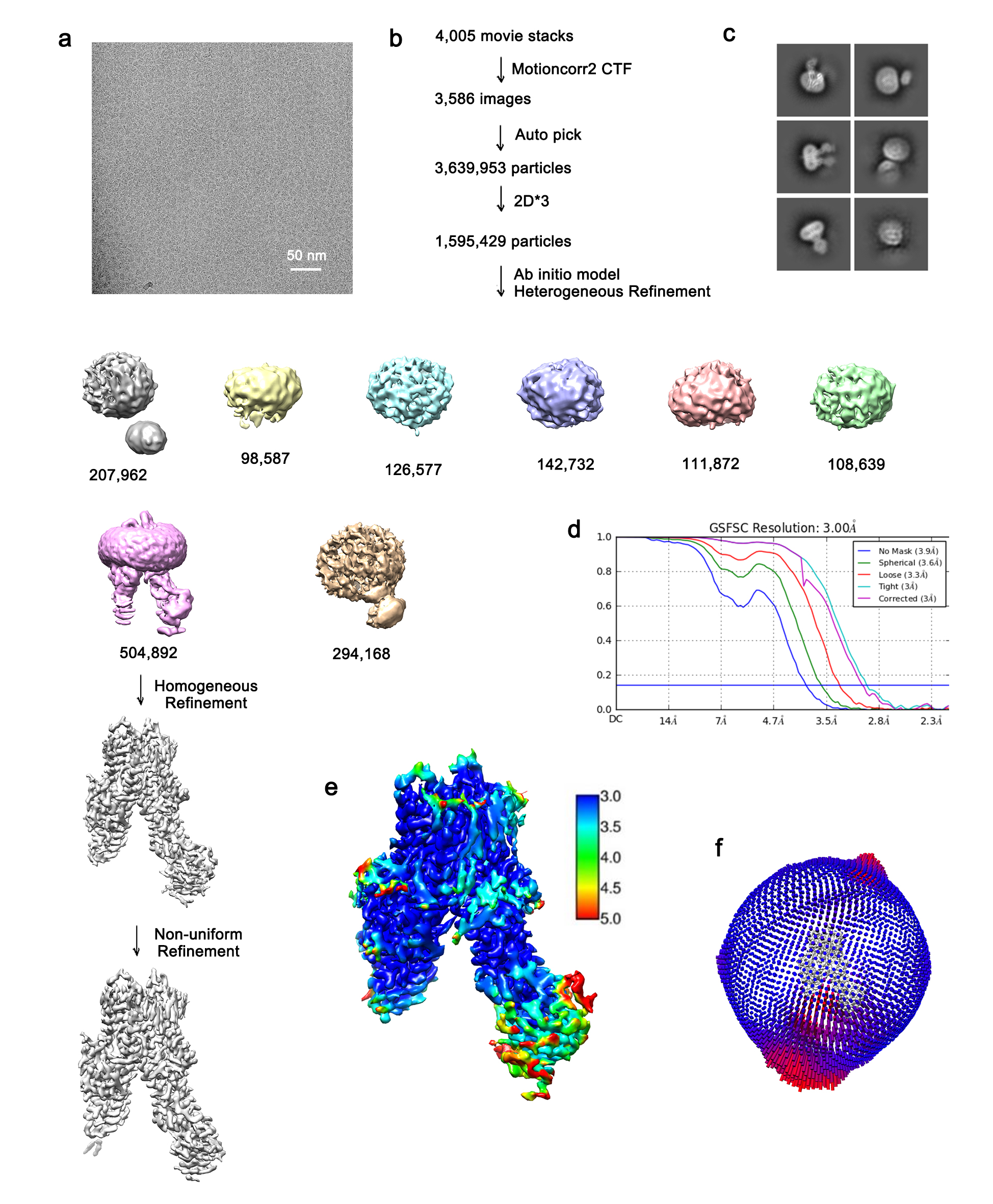

Supplement: Supplementary file 4 — Additional file 4: Fig. S4 Cryo-EM Data Collection and Processing of hABCC4 (MTX-Bound). Motion-corrected electron microscopy images. (b) Processing workflow of EM particle data. (c) Representative 2D classification of protein particle processing. (d) FSC data of the protein structure model reconstruction. (e) Resolution distribution of the density cloud. (f) Angular orientation results of the protein structure reconstruction. [file 13578_2025_1377_MOESM4_ESM.jpg]

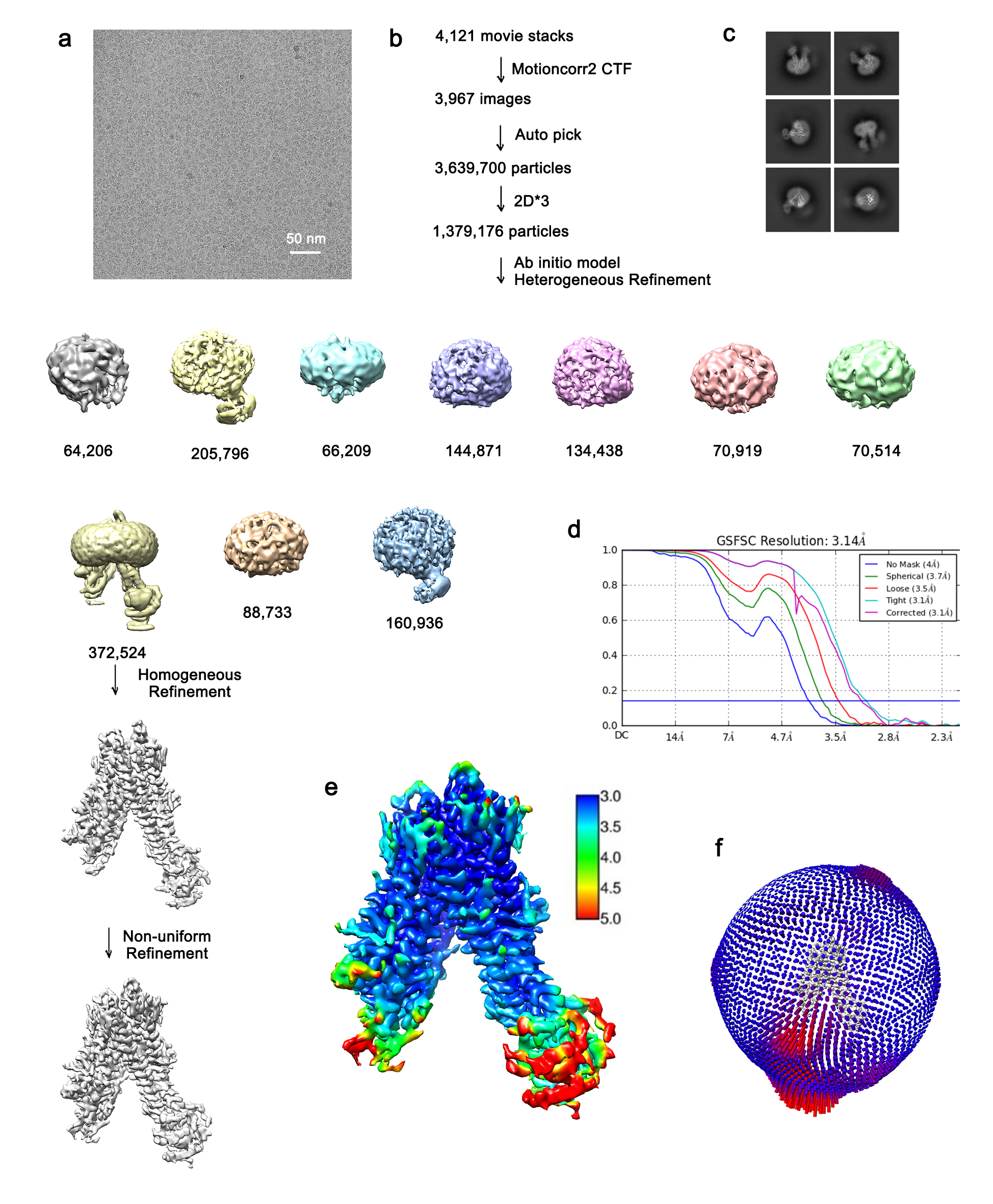

Supplement: Supplementary file 5 — Additional file 5: Fig. S5. Cryo-EM Data Collection and Processing of hABCC4 (PGE2-Bound). Motion-corrected electron microscopy images. (b) Processing workflow of EM particle data. (c) Representative 2D classification of protein particle processing. (d) FSC data of the protein structure model reconstruction. (e) Resolution distribution of the density cloud. (f) Angular orientation results of the protein structure reconstruction. [file 13578_2025_1377_MOESM5_ESM.jpg]
